# Design and Implementation of a New System for Large Bridge Monitoring—GeoSHM

**DOI:** 10.3390/s18030775

**Published:** 2018-03-04

**Authors:** Xiaolin Meng, Dinh Tung Nguyen, Yilin Xie, John S. Owen, Panagiotis Psimoulis, Sean Ince, Qusen Chen, Jun Ye, Paul Bhatia

**Affiliations:** 1 Faculty of Engineering, University of Nottingham, Nottingham NG7 2RD, UK; dinh.nguyen1@nottingham.ac.uk (D.T.N.); yilin.xie@nottingham.ac.uk (Y.X.); john.owen@nottingham.ac.uk (J.S.O.); panagiotis.psimoulis@nottingham.ac.uk (P.P.); sean.ince@nottingham.ac.uk (S.I.); 2 School of Geodesy and Geomatics, Wuhan University, Wuhan 430079, China; chenqs@whu.edu.cn; 3 UbiPOS UK Limited, Nottingham NG7 2TU, UK; jun.ye@ubipos.co.uk; 4 Geomatic Ventures Limited, Nottingham NG7 2TU, UK; paul.bhatia@geomaticventures.com

**Keywords:** structural health monitoring, GNSS, Earth Observation, long-span bridges, data strategy

## Abstract

Structural Health Monitoring (SHM) is a relatively new branch of civil engineering that focuses on assessing the health status of infrastructure, such as long-span bridges. Using a broad range of in-situ monitoring instruments, the purpose of the SHM is to help engineers understand the behaviour of structures, ensuring their structural integrity and the safety of the public. Under the Integrated Applications Promotion (IAP) scheme of the European Space Agency (ESA), a feasibility study (FS) project that used the Global Navigation Satellite Systems (GNSS) and Earth Observation (EO) for Structural Health Monitoring of Long-span Bridges (GeoSHM) was initiated in 2013. The GeoSHM FS Project was led by University of Nottingham and the Forth Road Bridge (Scotland, UK), which is a 2.5 km long suspension bridge across the Firth of Forth connecting Edinburgh and the Northern part of Scotland, was selected as the test structure for the GeoSHM FS project. Initial results have shown the significant potential of the GNSS and EO technologies. With these successes, the FS project was further extended to the demonstration stage, which is called the GeoSHM Demo project where two other long-span bridges in China were included as test structures. Led by UbiPOS UK Ltd. (Nottingham, UK), a Nottingham Hi-tech company, this stage focuses on addressing limitations identified during the feasibility study and developing an innovative data strategy to process, store, and interpret monitoring data. This paper will present an overview of the motivation and challenges of the GeoSHM Demo Project, a description of the software and hardware architecture and a discussion of some primary results that were obtained in the last three years.

## 1. Introduction

Long-span bridges are well known for their aesthetic and attractive appearance; featuring tall towers and large slender spans, they are considered to be a marvel of civil engineering. Moreover, they are key assets of infrastructure and transportation facilities, which provide connections between regions and facilitate regional cooperation as well as economic and social development of countries. A collapse or closure of bridges can lead to traffic chaos in regions, significant financial losses, and, in some cases, heavy casualties. For example, before the open of the Queensferry Crossing (September 2017), the Forth Road Bridge (FRB) was the major road link across the Fifth of Forth, Scotland; a single-lane closure of the FRB was estimated to cause a £650,000 loss daily. In 1993, the collapse of a 48 m central span of the Seongsu Bridge across the Han River in Seoul (South Korea) led to the death of 32 people and injuries to another 17 [1]. In 2007, the I-35W highway bridge over the Mississippi River in Minnesota (US) suffered a catastrophic failure, killing 13 people and injuring 145 others [2]. 

Learning from the noteworthy collapse of the Tacoma Narrows Bridge [3], long-span bridges have been designed with efficient aerodynamic characteristics to limit wind-induced effects. However, extreme events, such as spells of large temperature fluctuation, strong hurricanes, or storms and devastating earthquakes are more severe and occur at a higher rate, constantly presenting different threats to long-span bridges. Also, wind engineers are beginning to realise the importance of non-synoptic events that have not previously been featured in wind loading codes. Coupled with an increase in usage and demand, these inevitably accelerate the aging process and deterioration of structural elements, particularly for some bridges located in harsh environments. It is therefore important to thoroughly assess the safety, serviceability and sustainability of these structures during their operation, and hence SHM systems are being actively developed to fulfil this role. 

In many countries, such as China and Japan, it is now mandatory to implement Structural Health Monitoring (SHM) systems on long-span bridges for monitoring and evaluating their current health status. The Tsing Ma Bridge, which is a 1337 m long suspension bridge in Hong Kong, has been equipped with probably the most complete and sophisticated SHM system. This so-called Wind and Structural Health Monitoring System (WASHMS) consists of more than 800 sensors of different types permanently installed on the bridge, including Global Position System (GPS) sensors, accelerometers, and strain gauges [4]. Other examples are the Akashi Kaikyo Bridge [5], the Great Belt East [6], and the Sutong Bridge [7].

SHM systems on long-span bridges have greatly benefitted from the development of sensing technologies. Wireless sensor networks (WSNs) have attracted a lot of attention recently due to their unique advantages, which are low cost, high flexibility, and high efficiency. New advances of microelectromechanical systems (MEMS) technology make the deployment of dense and low power-consuming WSNs more feasible [8,9]. However, to fulfill this potential, there are weaknesses to this approach that need to be addressed, such as short lifetime, limited transmission distance, low transmission rate, and poor time synchronisation [10,11,12]. In addition, the development of fibre optic technologies has led to a new generation of sensors, which are known as fibre optic sensors (FOSs). These sensors are capable of providing reliable measurements of strain and temperature from multiple locations [13,14]. Many SHM systems on bridges have been equipped with these sensors, including the Tsing Ma Bridge [4], the Yonghe Bridge [15], the Ebian Bridge [16] and the Siggenthal Bridge [17]. FOSs are prone to breakage during installation and operation and are unreliable in case of poor contact between sensors and structures [17].

Global Navigation Satellite Systems (GNSS), in particular GPS technology, has been employed to monitor civil engineering infrastructures, including bridges for more than 20 years [18]. In a comparison with other traditional techniques, such as accelerometers, GNSS is superior in providing continuous and automated measurements and facilitating understanding of static profiles and dynamic behaviour of monitored structures [19,20,21]. Furthermore, the measurement of displacements facilitates the extraction of aerodynamic coefficients from full scale measurements. The deformation monitoring of bridges ranging from medium- to long-span suspension and cable-stayed bridges has greatly benefited from GNSS technologies [22,23,24,25]. However, this method is associated technical issues such as: influences the GPS satellite geometry on structural deformation monitoring [19], pervasive existence of dynamic multipath in the structural health monitoring measurements [26], low sampling rates [27], and communication stability in RTK-GPS positioning [28,29]. Moreover, the displacement measurement that is offered by GNSS brings about certain disadvantages in measuring high-frequency dynamic responses because of the limitations in sampling frequencies and the lack of resolution to measure very low displacements at high frequencies [18,30]. Thus, an integration of a dual frequency GNSS receiver and a tri-axial accelerometer has been focused on to improve the overall performance of the system in measuring low displacements and extracting dynamic information [30,31,32,33,34,35]. 

The evolution of sensing technologies has opened opportunities for the deployment of dense sensor networks on long-span bridges. Such systems can generate a large amount of data, which imposes a further major challenge to SHM communities, that there is a lack of a robust data interpretation technique to obtain comprehensive information on the structural health status of long-span bridges. Depending on the presence or absence of physical models, data interpretation methods are classified into model-based and model-free methods. Their advantages and disadvantages are discussed in detail in [36]; both of them are complementary, depending on context and application.

The aim of model-based methods is to develop a reliable behaviour model which can predict structural responses; finite-element models are the most common approach. Differences between predictions and actual responses are classified as system changes or damages of structures [37,38]. Behaviour models can also be used to study different failure scenarios, to estimate long-term performance of structures, and to evaluate the serviceability performance, which are very beneficial for structural management [39]. However, the development of such behaviour models is very expensive and they may not reflect real structures due to the complexity of long-span bridges [40]. Further studies to improve this approach and minimise uncertainties within behaviour models have been conducted [41,42]. 

Model-free methods are also known as the data-driven approach, which involves the direct analysis of time series without knowledge of the geometry and materials of structures. Their target is to detect changes in signals, which are considered to correspond to anomalous behaviour of structures under external excitation [43,44]. Without the need to perform heavy computational simulation, these methods are generally faster and more appropriate for continuous monitoring of structures. Wavelet-based, autoregressive-based, and principal-component-analysis-based methods are typical examples of this approach [45,46]. With the development of machine learning and pattern recognition, this approach has extended to learning algorithms, such as artificial neural network, support vector machines, and random forest [47]. The success of these methods depends greatly on the availability of historical monitoring data to establish signature behaviours of structures, which is known as a training process. In addition, the selection and evaluation of damage-sensitive features are important; these processes primarily depend on data interpretation methods and monitored structures. Some features such as modal frequencies are significantly influenced by environmental and operational conditions including air temperature and traffic [48]. More importantly, outliers can appear in signals due to technical issues of sensors or errors during data transmission and processing. Outliers potentially not only mask the signature behaviour of a structure, but also are potentially mistaken as damage; therefore, it is important to identity and exclude them when interpreting monitoring data [49,50].

## 2. Motivation of Structural Health Monitoring of Long-span Bridges (GeoSHM) R&D

In contrast to other mechanical engineering structures, civil engineering infrastructure, including long-span bridges, are classified as large scale structures; an effective SHM therefore relies on a dense sensor network. It can be beneficial, providing bridge operators and engineers a detailed understanding and information of structural performances of bridges. However, as pointed out in Section 1, due to the nature of monitored quantity, such a sensor system not only incurs large expense but also generates a huge amount of data, which easily becomes a burden in terms of data analysis, interpretation and storage. More importantly, processing large data sets can mask important features in the data, which are potentially associated with structural damages or extreme environmental or operational conditions. Also, the use of denser sensor systems can be inefficient since the configuration of sensor systems is not appropriate to detect damage that is most likely to occur on a structure [51]. 

The damage detection in the SHM of long-span bridges is very challenging since there is no comprehensive definition of damage; damage is often unique and bridge specific. Many bridges are one-of-a-kind structures and are susceptible to different types of damage at different locations. For this particular reason, the establishment of thresholds and baselines to assess the safety, serviceability, and sustainability of bridges is not reliable in most cases. They do not represent either the current structural characteristics or the actual external loading on bridges. In addition, as non-linear structures, long-span bridges give rise to monitoring data that are non-linear and multivariate, causing difficulties to understand and extract relationship between data acquired from different sensors. This issue is exacerbated by the presence of outliers in data and the influence of environmental and operational conditions.

**Figure 1:**
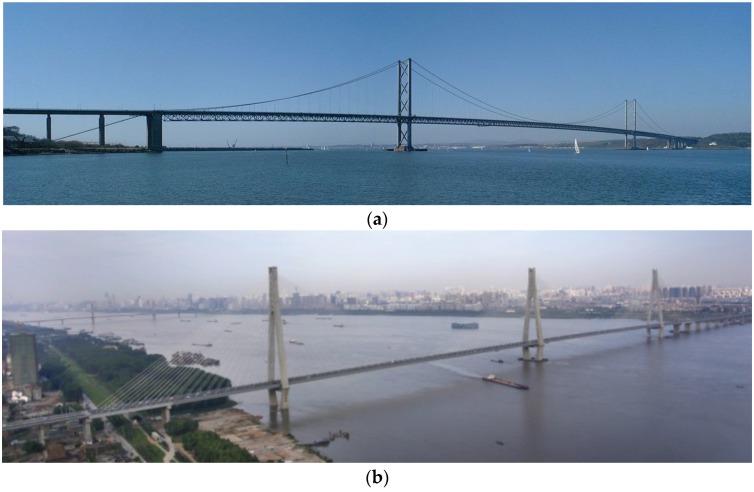

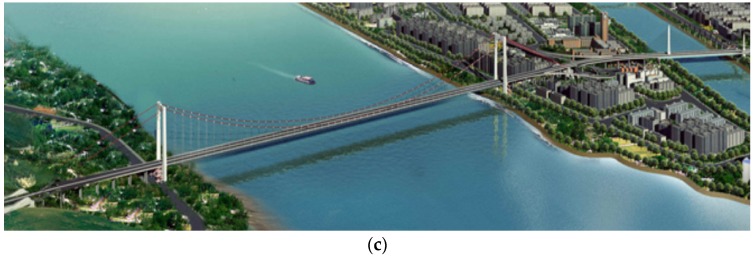
Three test structures in the Structural Health Monitoring of Long-span Bridges (GeoSHM) Demon Project: (**a**) the Forth Road Bridge (FRB) in Scotland, UK (Image from Wikicommons. Author: Klaus with K.); (**b**) the Erqi Bridge in Wuhan, China; and, (**c**) the Zhixi Bridge in Yichang, China.

Following the success of the FS study, the GeoSHM Demo Project was funded by the European Space Agency (ESA) to fully address these limitations and to deliver a complete SHM system for long-span bridges. Three long-span bridges in the UK and China were selected to be the test structures (Figure 1); one of them is the Forth Road Bridge (FRB). The GeoSHM Demo Project aims to develop an integrated sensor system featuring the use of remote sensing technologies—GNSS, and, in particular, the state-of-the-art Earth Observation technologies. The former is primarily used to measure bridge deformation, which is complemented by other low-cost sensors, such as accelerometers and inclinometers. For the latter, this technology is capable of identifying subsidence surrounding bridge sites, which is then interpreted to study ground movements around the foundations of bridges and to ensure the integrity of structures. In addition to the integrated sensor system, the key aim of this project is the development of an innovative data strategy. This is hereafter referred as the GeoSHM Data Strategy, which details methodologies and strategies to extract information and evaluate the structural health status of bridges, aiding bridge operators in their decision making process.

In this paper, we report on the current progress of the GeoSHM Demo Project, focusing on the development of the GeoSHM system and the application on the FRB together results of preliminary data interpretation. In Section 1 and Section 2, the background and challenges in the SHM of long-span bridges are presented, which sets the motivation for the GeoSHM Demo Project. Section 3 is devoted to describe five key modules of the GeoSHM system to address and fulfill user and system requirements. In Section 4, some important observations regarding structural responses due to effects of wind, traffic and temperature as well as ground movements after approximately three years of deploying sensors on the FRB will be discussed, which will show the potential of the GeoSHM system in informing bridge owners on the performance and health status of the bridge, thus supporting their decision making.

## 3. GeoSHM System Architecture

**Figure 2:**
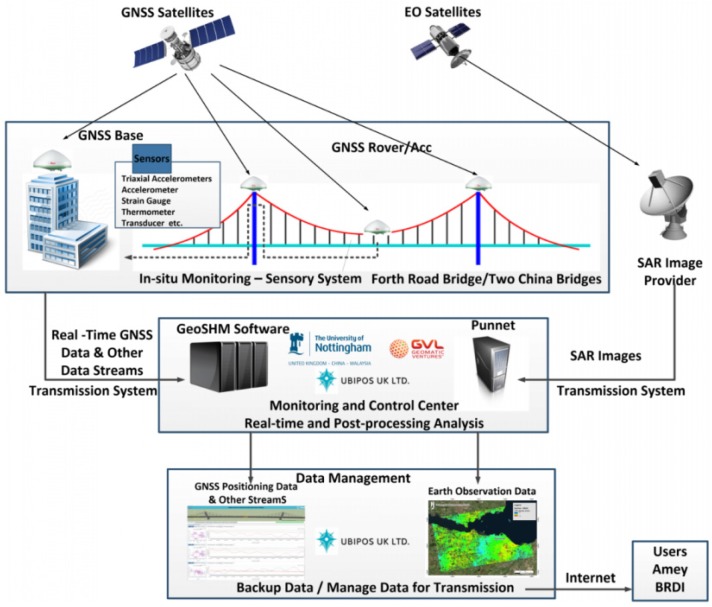
The overall GeoSHM system architecture.

**Figure 3:**
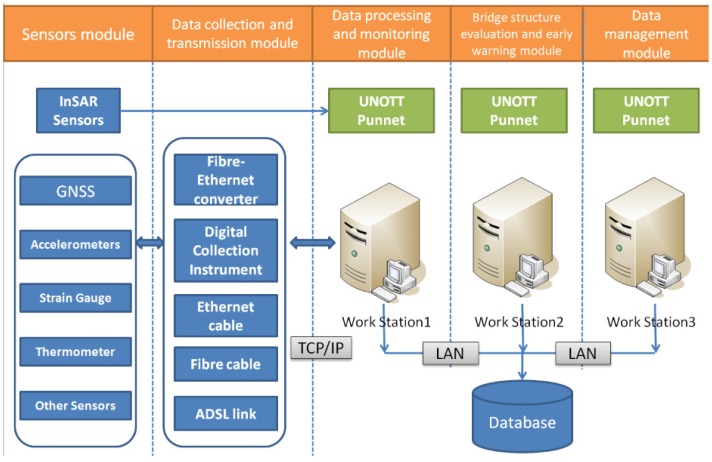
The relationship of GeoSHM sub-systems and data flow.

The overall GeoSHM system architecture for the FRB is shown in Figure 2, which can be divided into five sub-systems: the sensor module, data collection and transmission module, data processing and monitoring module, bridge structure evaluation and early warning module and data management module. Their relationship and components are summarised in Figure 3. In this figure BRDI is an abbreviation of the China Railway Major Bridge Reconnaissance and Design Institute Co., Ltd. (Wuhan, China), a key partner of GeoSHM team. 

### 3.1. Sensor Module

**Figure 4:**
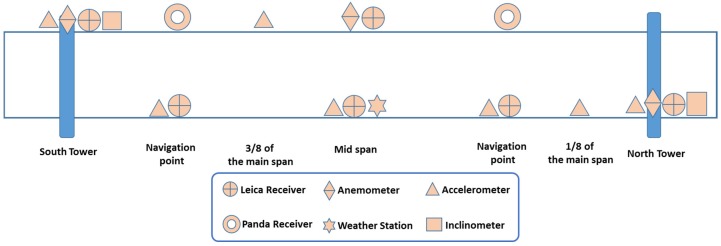
Locations of sensors to be installed in the GeoSHM Demo project.

The GeoSHM sensor module comprises a range of different sensors to monitor not only the structural responses of the bridge (displacement, acceleration, inclination, and stresses), but also external loads that are applied on the bridge (wind load and traffic weight) and the short- and long-term environmental effects (temperature, weather conditions, and ground movements). Table 1 summarises details of sensors deployed in this GeoSHM Demo Project as well as their sampling rates; their locations on the FRB are described in Figure 4. 

The GNSS techniques that are offered by the use of high-performance Leica GNSS receivers and low-cost Panda GNSS receivers is the key of the sensor module, providing both the static profile and dynamic behaviour of the bridge. The use of high-performance receivers together with low-cost receivers is one of the system requirements to develop a cost-effective sensor module. On the main span of the FRB, three pairs of GNSS receivers together with three low-cost tri-axial Sherborne accelerometers are installed at the mid-span and two navigational points. This combination facilitates an integration of GNSS and acceleration data to achieve high-accuracy bridge deformation. Two further accelerometers are installed at 1/8 and 3/8 of the main span, which provide additional data to identify modal frequencies and vibrational mode shapes of the FRB. For the two main towers, their deformation is important since it is highly correlated with the deformation of the main span. A combination of a GNSS receiver and a tri-axial accelerometer is placed at the top of each tower. Also, an inclinometer is installed to provide an indication of averaged deformation at the top of the towers. There are three anemometers installed on the FRB; one is at the mid-span while the other two are on the top of the two main towers, which facilitate a detailed correlation study of the wind load on the FRB. Also, a MET (mini weather) station is placed at the mid span, and it measures air temperature, pressure, and humidity. Moreover, the state-of-the-art EO technology is employed in the GeoSHM Demo Project, providing valuable data on the subsidence in Edinburgh, which can adversely affect the foundation of the main towers and the integrity of the bridge.

### 3.2. Data Collection and Transmisstion Module

**Figure 5:**
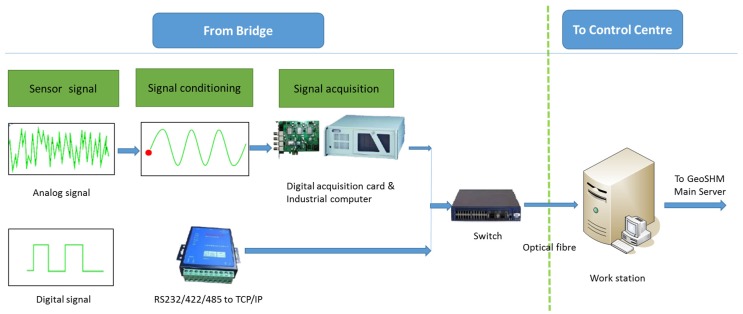
Schematic of GeoSHM data collection and transmission module.

Figure 5 describes the data acquisition and transmission module that is deployed at the FRB. This module features the use of an optical fibre system to communicate with sensors and transmit their data to a server securely located in the control centre on site. This server offers bridge operators access to the monitoring data with the purpose of real-time monitoring, data download for further analysis and routine report generation. More importantly, this is also a back-up server to temporarily store raw sensor data in case there is an interruption in the communication between the bridge site and the main GeoSHM server located at the University of Nottingham. Due to the combination of a number of sensors and high sampling rates, the back-up server is designed to store raw sensor data for approximately one month, which is sufficient in case of potential communication interruption. 

### 3.3. Data Processing and Monitoring Module

**Figure 6:**
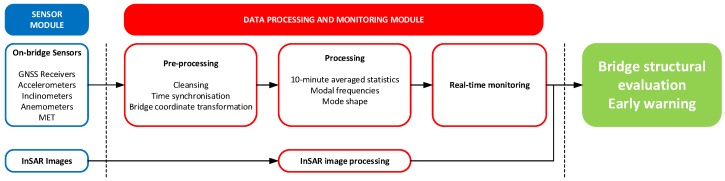
Key functionalities of GeoSHM data processing and monitoring module.

The data processing and monitoring module is deployed primarily inside the main GeoSHM server located at University of Nottingham and consists of two units: the pre-processing and the post-processing units (Figure 6). The key functionality of the former is to synchronise data from all of the sensors based on GPS time and to identify and remove outliers. In addition, the pre-processing unit is designed to convert raw sensor data into appropriate monitoring data; for example, after pre-processing, the cleansed GNSS data will be transformed to bridge deformations defined in the bridge coordinate system. Outputs of the pre-processing unit will be transferred into the post-processing unit where 10-min statistical averages of features relating to bridge deformation, external loading and environmental effects will be evaluated, such as mean and standard deviation (std) of the bridge deformation at the mid-span, average slopes of the main towers, peak factors of the wind, averaged air temperature, etc. These low-level features are clearly defined in the GeoSHM Data Strategy. To complete this GeoSHM Smart Data Analysis, an automated and sophisticated system identification algorithm is included to estimate modal frequencies and shapes of the vibrational modes by using GNSS and acceleration data. In addition, this unit includes a real-time or near real-time monitoring function where these 10-min averaged features are used to provide bridge operators with a comprehensive visualisation of bridge deformation under wind loads and other environmental and operational factors. It is noticed that the InSAR image processing is carried on a monthly basis due to its high computational demand and low sampling rate. This is reasonable since subsidence occurs at a much longer time scale when compared to other external effects. 

### 3.4. Bridge Structure Evaluation and Early Warning Module

**Figure 7:**
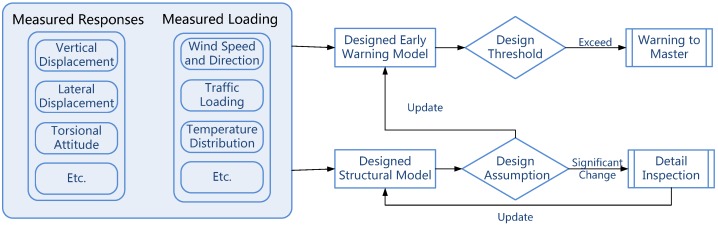
The flow chart of building block for GeoSHM Module Four.

Figure 7 represents the design and implementation of this module based on the warning strategy that is set out in the GeoSHM Data Strategy. The functionality of this module is dependent on the thresholds and baselines initially established based on historic monitoring data and the experience of bridge operators. An iterative updating mechanism is employed to continuously refine the thresholds and baselines so that they can reflect the current behaviour of the bridge. Once the measured bridge responses exceed these thresholds and baselines, a warning will be raised to indicate that the structural behaviour of the bridge is abnormal. A higher-level structural evaluation mechanism will be then employed to further investigate this alarm and to clarify whether it is resulted from changes in operational and environmental conditions or it is related to system changes of the structure or failures of structural members. This mechanism also involves a rolling update of a structural model using modal parameters that are extracted from deformation data. The refined model facilitates simulations of dynamic behaviour of the structure and assessment of risks associated with structural damage, which aid bridge operators with their management activities. As for the InSAR image processing, once outcomes are available, they will be applied to the structural model in order to evaluate effects of the long-term ground movement on the rigidity of the structure.

### 3.5. Data Management Module

The data management module comprises protocols and policies to manage raw data and outcomes of the GeoSHM Smart Data Analysis. It is also capable of generating routine reports to bridge operators and controlling data access for different groups of users. This module is designed to be adaptable to the potential changes of the modules mentioned previously, as well as an increase in the number of sensors and features to be extracted. The archiving strategy set out in the GeoSHM Data Strategy is implemented to store all 10-min averaged features in the GeoSHM database. In addition, raw pre-processed data is stored to provide opportunity to refine the main archive as knowledge of the bridge behaviour develops. This raw data is compressed and stored for a limited period time to avoid compromising storage spaces in the main GeoSHM server. 

## 4. Results and Discussion

### 4.1. Status of Sensor System and Software Development

**Figure 8:**
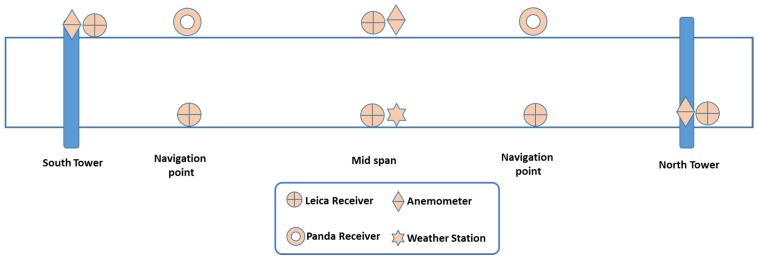
Current distribution of sensors on the FRB.

Figure 8 describes the current status of the sensor module on the FRB. Approaching the end of the second phase of the sensor installation, ten GNSS receivers, including eight high-performance Leica receivers and two low-cost Panda receivers, were installed on the bridge; each Panda receiver is located at one navigational channel point (i.e., one-quarter of the main span). In addition, there is one MET station that is installed at the mid span and three anemometers located at the mid-span and the top of two main towers.

**Figure 9:**
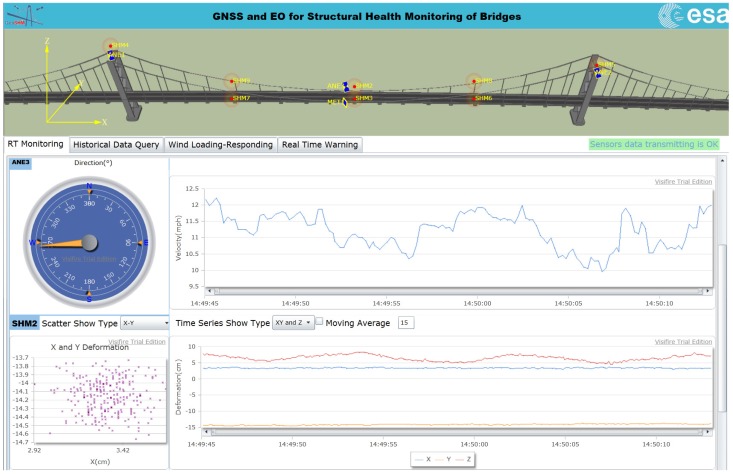
GeoSHM web application.

Regarding the software development, a GeoSHM web application is being developed, as shown in Figure 9. This provides a platform facilitating communication between users and the GeoSHM system. Depending on data policies, different groups of users will be able to access certain functionalities of the web application comprising real-time monitoring, real-time warning, and historical data query. Some results that were obtained from the web application will be discussed further in Section 4.2.

### 4.2. Preliminary Analysis of Bridge Deformation

In addition to the real-time monitoring, the GeoSHM web application offers users an option to interrogate the GeoSHM database of the 10-min averaged features average statistics. This function is very useful for bridge operators and engineers to understand historical and long-term structural response of the bridge, as well as to perform further inspection of bridge responses once system changes or failures of structural members are detected. Moreover, the assessment of 10-min average statistics of bridge responses, natural frequencies, wind speeds, and temperature is important to establish short- and long-term trends or baselines, as well as thresholds. They are essential to define normal structural behaviour of the FRB under different environmental and operational conditions, which facilitate the development of bridge structural evaluation and early warning algorithms. In this section, some results of this assessment will be presented and discussed. Figures that are included hereafter can be generated automatically by the GeoSHM web application upon request. However, the authors reproduce them here by using MATLAB for high resolution presentation.

**Figure 10:**
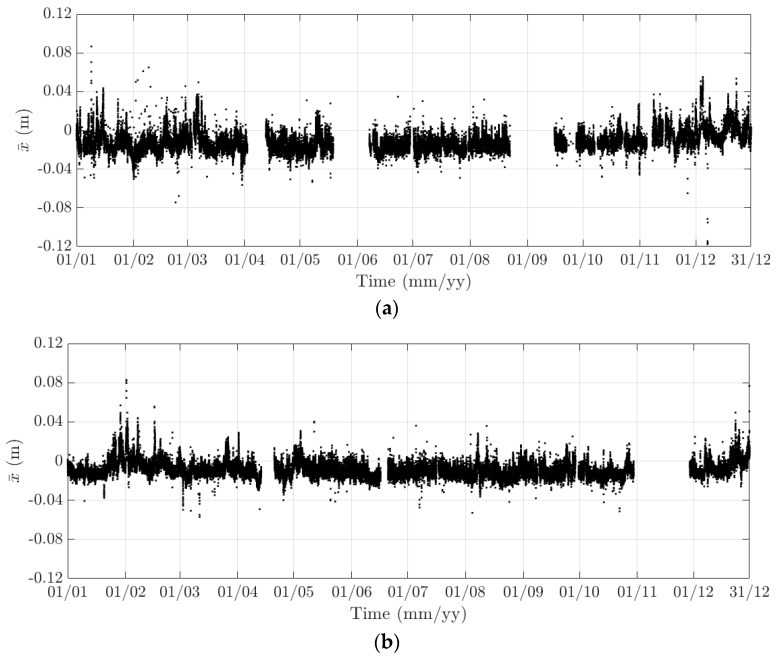
Variation of the 10-min mean of the longitudinal response at the mid span of the FRB in (**a**) 2015 and (**b**) 2016.

**Figure 11:**
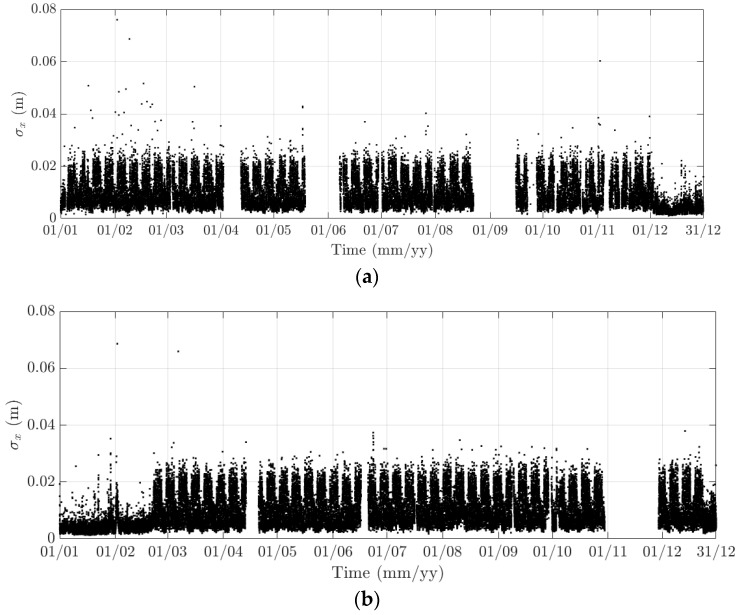
Variation of the 10-min standard deviation of the longitudinal response at the mid span of the FRB in (**a**) 2015 and (**b**) 2016.

**Figure 12:**
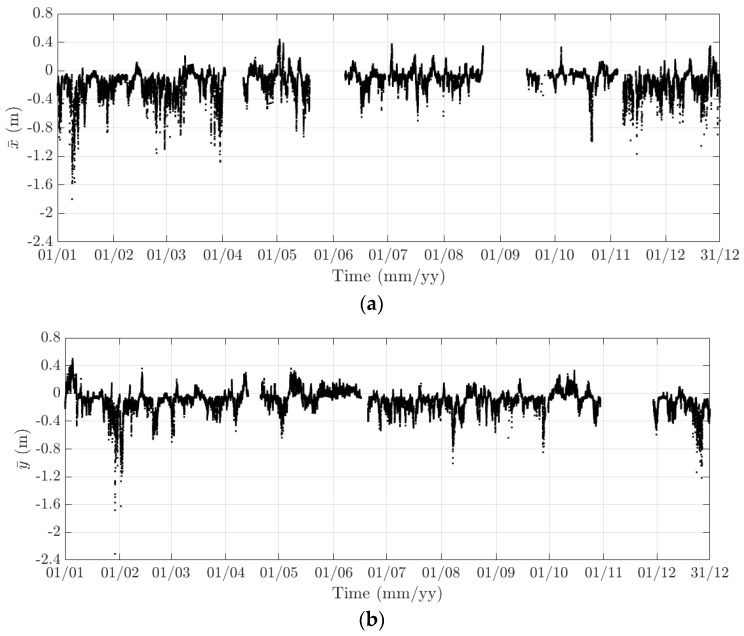
Variation of the 10-min mean of the lateral response at the mid span of the FRB in (**a**) 2015 and (**b**) 2016.

**Figure 13:**
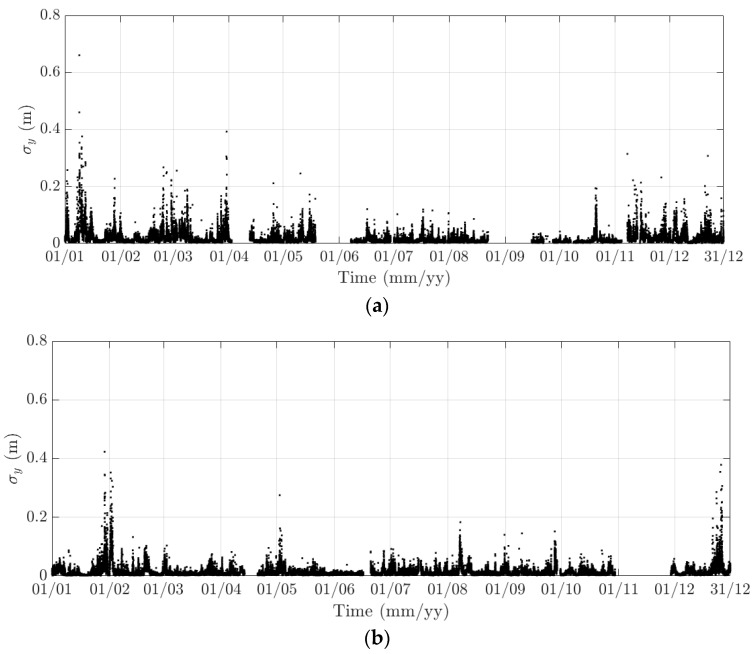
Variation of the 10-min standard deviation of the lateral response at the mid span of the FRB in (**a**) 2015 and (**b**) 2016.

**Figure 14:**
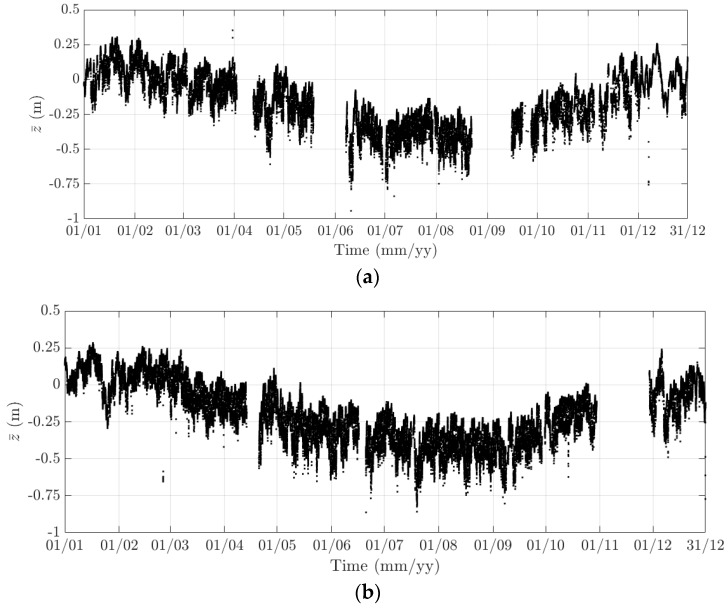
Variation of the 10-min mean of the heaving response at the mid span of the FRB in (**a**) 2015 and (**b**) 2016.

**Figure 15:**
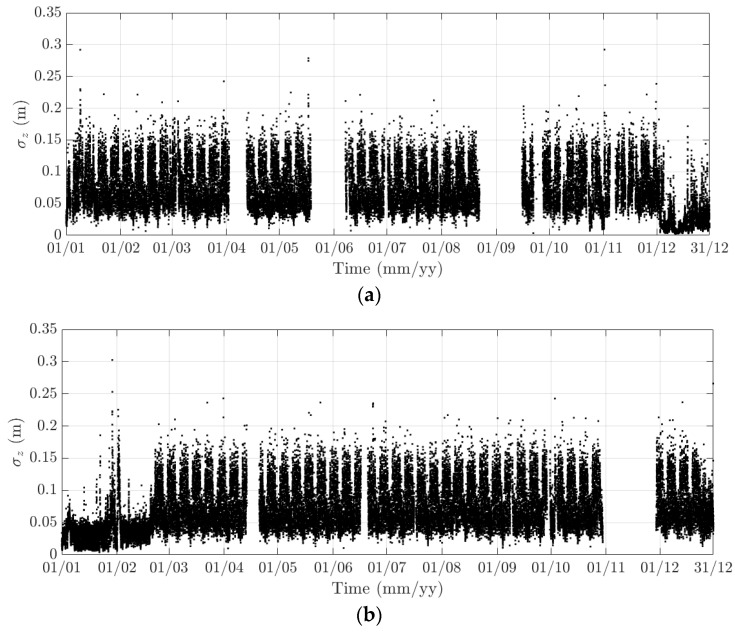
Variation of the 10-min standard deviation of the heaving response at the mid span of the FRB in (**a**) 2015 and (**b**) 2016.

**Figure 16:**
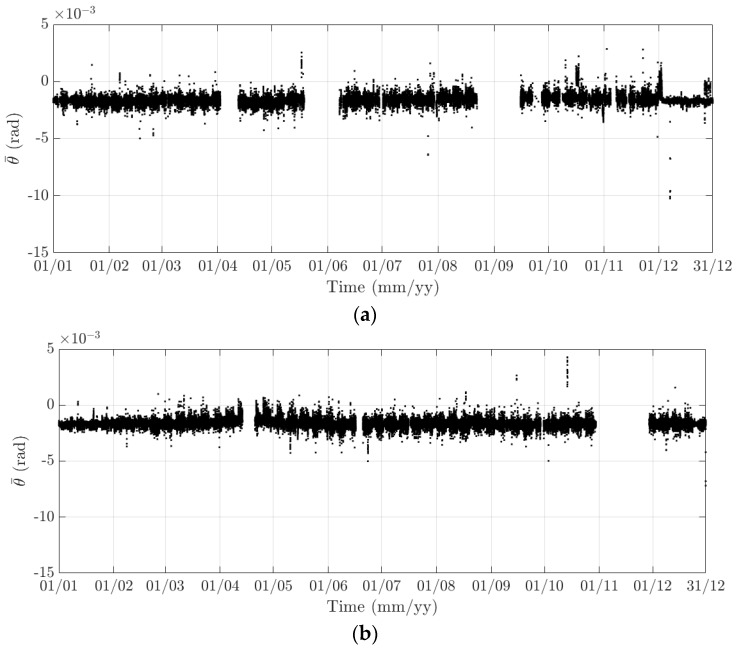
Variation of the 10-min mean of the torsional response at the mid span of the FRB in (**a**) 2015 and (**b**) 2016.

**Figure 17:**
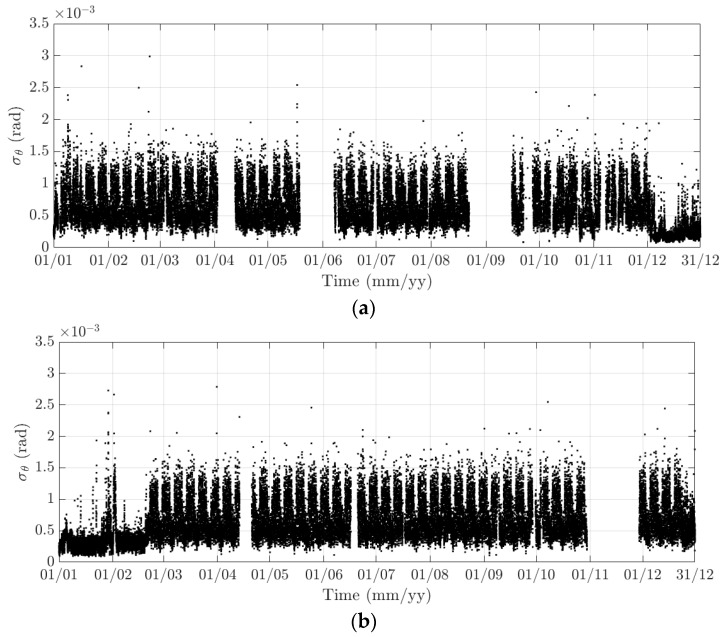
Variation of the 10-min standard deviation of the torsional response at the mid span of the FRB in (**a**) 2015 and (**b**) 2016.

**Figure 18:**
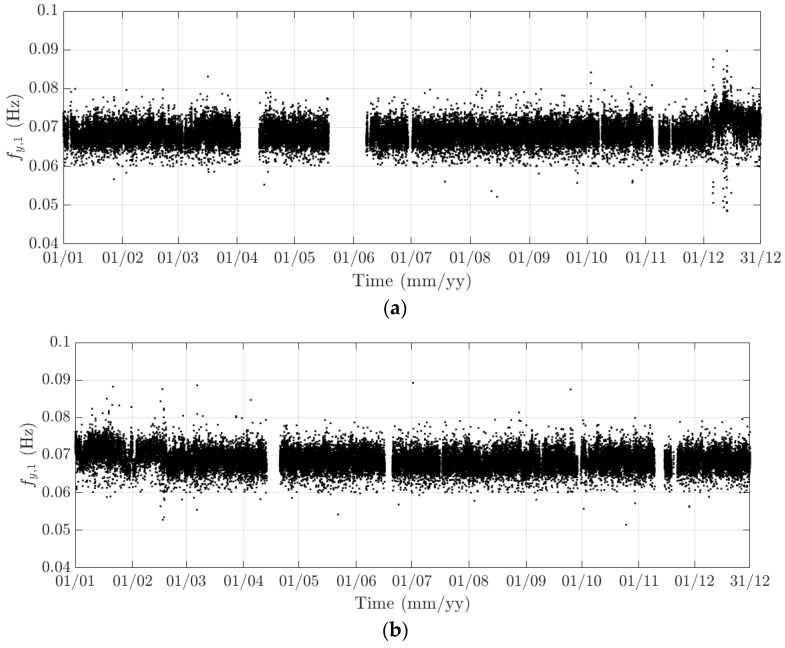
Variation of the 10-min natural frequency of the first lateral model in (**a**) 2015 and (**b**) 2016.

**Figure 19:**
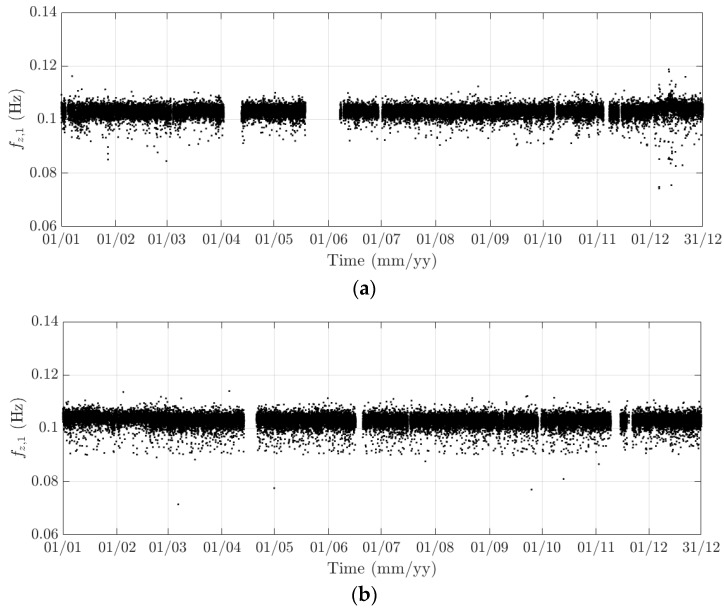
Variation of the 10-min natural frequency of the first heaving model in (**a**) 2015 and (**b**) 2016.

**Figure 20:**
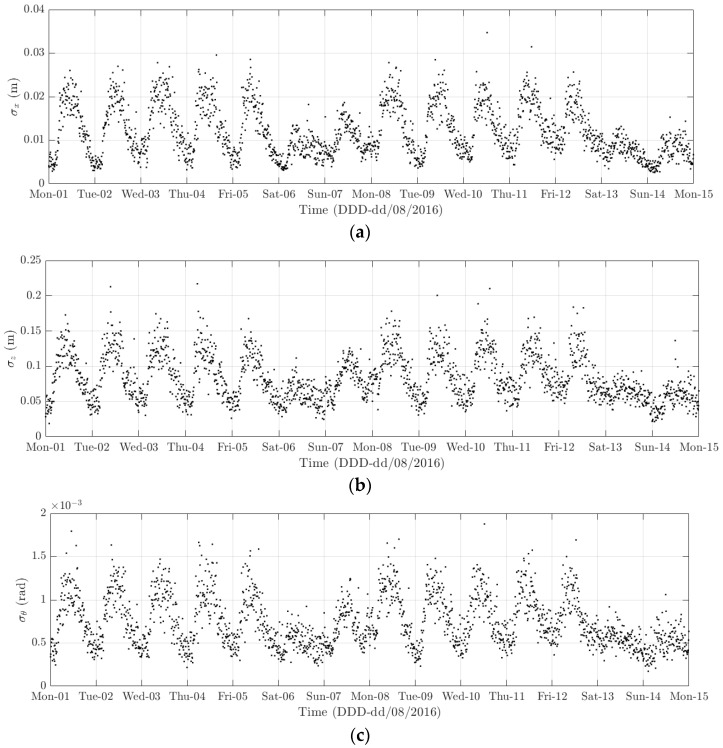
Variation of the 10-min standard deviation of (**a**) the longitudinal deformation; (**b**) heaving deformation; and, (**c**) torsional deformation from 1 to 14 August 2016.

**Figure 21:**
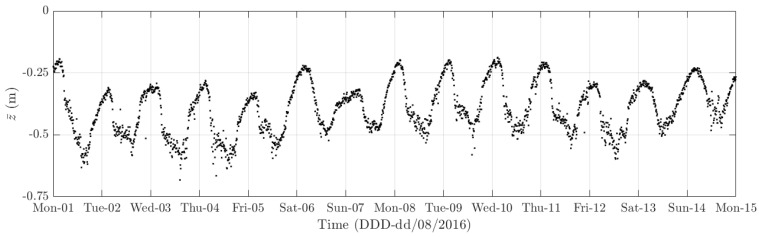
Variation of the 10-min mean of the heaving deformation from 1 to 14 August 2016.

**Figure 22:**
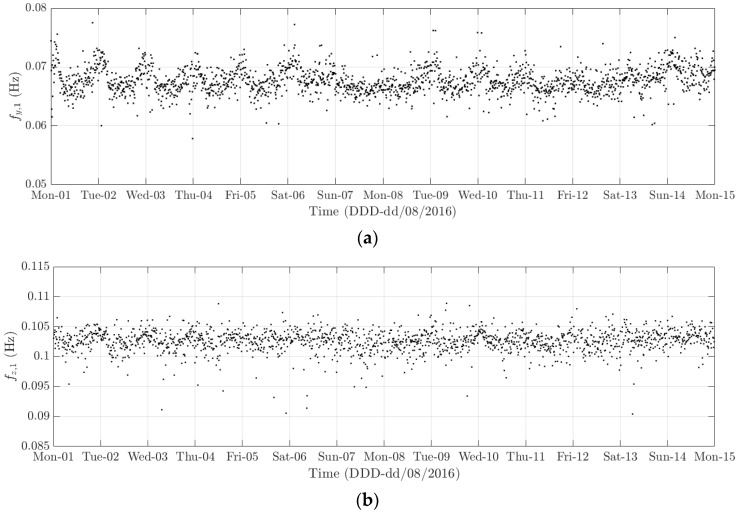
Variation of the 10-min natural frequencies of (**a**) the first lateral mode and (**b**) the first heaving mode from 1 to 14 August 2016.

Here, 10-min mean and standard deviation of bridge responses at the mid-span of the FRB are primarily considered, together with their dependence on the wind, air temperature, and traffic. Bridge responses comprise the longitudinal (along the *x* axis), lateral (along the *y* axis), heaving (along the *z* axis), and torsional (around the *x* axis) one. Values of the 10-min mean provide some indication of long-term deformation of the bridge while the 10-min standard deviation is representative of the dynamic component of bridge responses. By analysing monitoring data in 2015 and 2016, periodicity in bridge responses and natural frequencies is revealed, which can be classified into diurnal, weekly, and annual cycles. Some of these observations are very similar to the results that were obtained from the SHM system installed on the Tamar Bridge, which is a 560 m suspension bridge located in Plymouth, UK [47,48]. There are a number of occasions when bridge responses and natural frequencies did not follow these patterns, which will be explained further in this section. It should be noticed that Figure 10, Figure 11, Figure 12, Figure 13, Figure 14, Figure 15, Figure 16, Figure 17, Figure 18 and Figure 19 present these 10-min average features in 2015 and 2016, while in Figure 20, Figure 21 and Figure 22, some selected features are shown in a short duration (1 August to 14 August 2016) for detailed analysis.

The diurnal cycle can be observed very clearly when investigating 10-min standard deviation of the longitudinal response (Figure 12), the heaving response (Figure 15), and the torsional response (Figure 17). Figure 20 shows the detailed variation of these 10-min average statistics in the duration from 1 to 14 August 2016. It is clear that during the daytime, values of the standard deviation is larger than during the nighttime. An increase in traffic occurs around 03:00 to 04:00 and leads to larger dynamic responses; after 15:00, less traffic is on the bridge, causing a gradual drop of dynamic responses. Since there is more traffic present on the bridge on weekdays, the variation of the standard deviation is consequently larger when compared to that at weekends. In addition, the diurnal cycle can be revealed by studying the 10-min mean of the heaving deformation (Figure 14), which represents the sagging at the mid span of the FRB. As shown in Figure 21, the amount of sagging is larger during the daytime than that in the nighttime. However, natural fluctuations in the air temperature make this diurnal pattern less visible. Since there is less traffic present on the bridge at weekends, the sagging at the mid span is found to subsequently reduce. These diurnal cycles are also observed on the 10-min natural frequencies of the first lateral and heaving modes (Figure 18, Figure 19 and Figure 22). During the day, an increase in the air temperature and additional mass due to traffic cause these frequencies to reduce by 7% and 2%, respectively, during the daytime; this pattern is less pronounced at weekends (Figure 22).

The weekly cycle is also very noticeable when studying the 10-min standard deviation of bridge responses (the longitudinal, heaving and torsional responses) and the natural frequencies (Figure 20 and Figure 22). This pattern comprises five weekdays featuring large variation in dynamic responses and natural frequencies and a weekend when these variations diminish significantly. This is caused by the difference in the amount of traffic on the bridge between weekdays and weekends. The weekly cycle can also be observed in the 10-min mean of the heaving deformation; however, natural long-term fluctuations in the air temperature can make it less apparent (Figure 21). 

In 2015 and 2016, there are a number of occasions where the bridge responses and natural frequencies departed from these trends: (i) early in January 2015; (ii) from December 2015 to February 2016 and (iii) later in December 2016. During these periods, changes in the standard deviation during one day are less pronounced and the natural frequencies increase significantly, particularly in (ii). By analysing the variation of the air temperature and traffic in these events, it was found that they were caused by a reduction in traffic on the bridge. If (i) and (iii) were public holidays (Christmas and New Year holidays), (ii) was associated with the fracture of the North East Truss end link, which led to the closing of the bridge and traffic restriction. This incident is less visible on the 10-min mean of the heaving deformation, but caused large variation on the 10-min mean of the longitudinal and torsional deformation, even though their long-term variation is minimal (Figure 10 and Figure 16). 

The 10-min mean of the heaving deformation clearly exhibits an annual cycle (Figure 14). From January to August, due to an increase in the air temperature, the sagging of the mid span of the FRB steadily increases and eventually reaches an average of approximately 0.4 m. After August, as the ambient temperature starts to decrease, the FRB gradually returns to its original position. The annual cycle is not present in other components of the bridge response or natural frequencies. 

**Figure 23:**
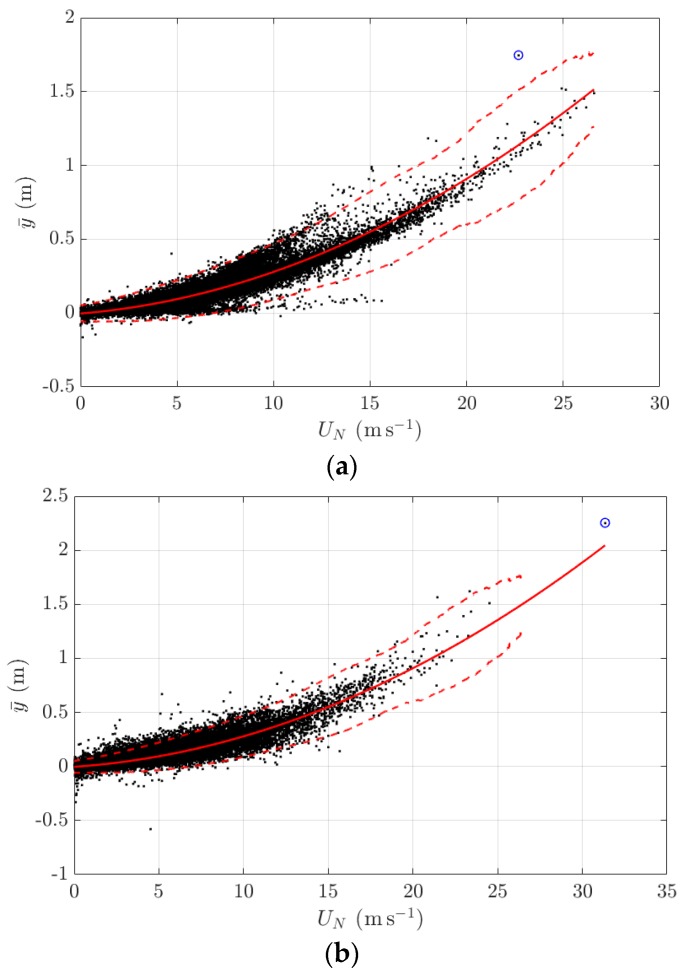
Variation of the mean lateral displacement at the mid span against the normal component of the mean wind speed in a comparison with the developed quadratic curve and thresholds; (**a**) 2015 and (**b**) 2016; extreme events are highlighted by blue circles.

The 10-min mean and standard deviation of the lateral deformation is largely influenced by the wind; therefore data appears to be more random and no clear short- and long-term trends are visible (Figure 12 and Figure 13). Instead, investigating plots of the wind loading response as shown in Figure 23 revealed the quadratic relationship between the mean lateral deformation and the normal component of the mean wind speed. Lower and upper thresholds are included in Figure 23 and are defined as ±3 σ with σ being the standard deviation of a data sample in a 3 m s^−1^ window of the normal component of the mean wind speed. It is obvious that majority of data lie within these two thresholds; some data points (highlighted by blue circles on Figure 23) are classified as extreme events, where wind speeds are very high or the mean lateral response significantly deviates from the established quadratic curve and/or exceed the upper threshold.

The review of the monitoring data in 2015 and 2016 has established some signature structural behaviours of the FRB under external excitation. The influence of the air temperature and traffic leads to some repetitive patterns of bridge responses and modal frequencies. Depending on causes and time scales, these patterns are categorised into diurnal cycles, weekly cycles, and annual cycles. Changes in operational conditions, which are due to either closures of the FRB or public holidays, were found to cause some deviations from these periodic patterns. Also, the baseline together with the lower and upper thresholds of wind-induced responses are defined and are essential to ensure normal structural responses of the FRB at high winds. As part of the development of the GeoSHM data strategy, the establishment of these signature behaviours of the FRB are very important to assess the monitoring data in subsequent years to identify system changes of the structure and their causes. 

### 4.3. Preliminary Analysis of InSAR Images

In this section, some preliminary results of the InSAR image processing will be presented covering the surrounding area of the FRB (Scotland, UK) and the Erqi Bridge (Wuhan, China). These results were prepared by the Geomatic Ventures Limited, which is a sub-contractor of the GeoSHM Demonstration Project and spin-out company of University of Nottingham during delivery of the GeoSHM FS Project. They are responsible for the development and implementation of the EO technology and InSAR image processing.

**Figure 24:**
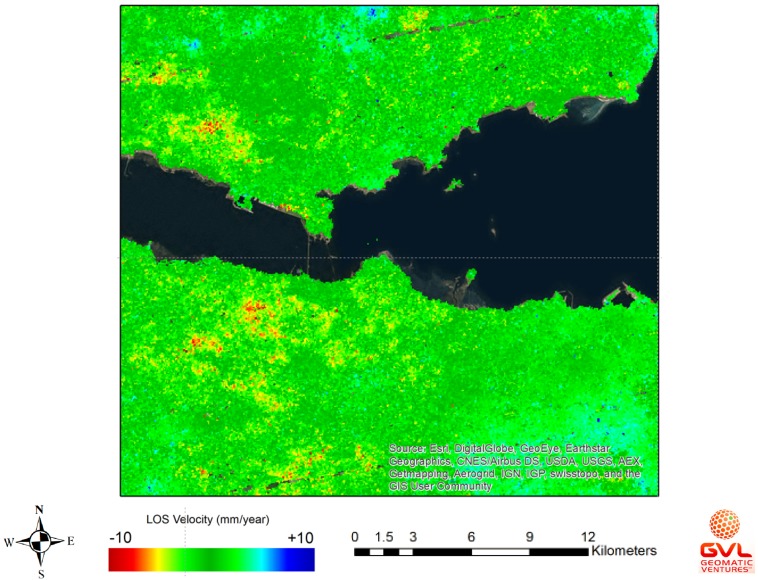
InSAR image processing of the area around the FRB, Scotland (October 2017).

**Figure 25:**
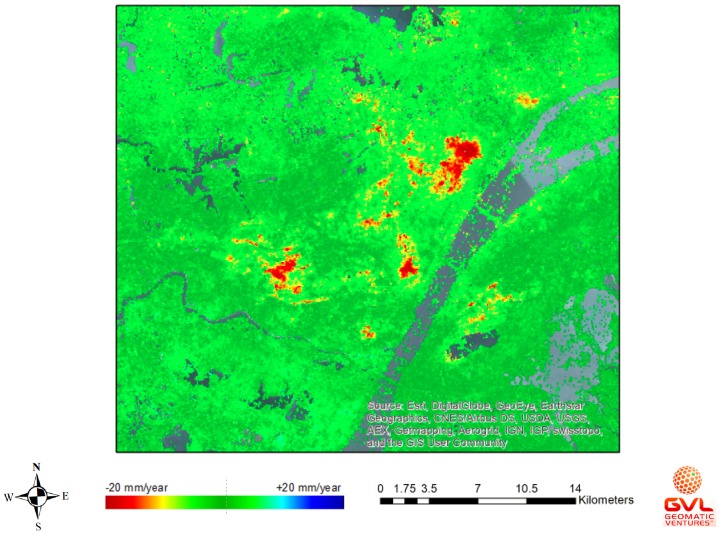
InSAR image processing of the area around the Erqi Bridge, China (September 2017).

As shown in Figure 24, the subsidence movement of the area that is surrounding the FRB is negligible. Some downward movements at a rate of about 5 mm year^−1^ were observed to occur at approximately 2 km away from the FRB; however, their effects were minimal. As for the Erqi Bridge (Wuhan, China), Figure 25 shows the existence of significant subsidence movements at a maximum rate of 20 mm year^−1^ and at a distance of 1 km away from the bridges. These have tendency to spread towards the bridge sites endangering the structural integrity of the bridge.

## 5. Conclusions

With more and more long-span bridges being built in developing countries, especially in China, and existing bridges in developed countries suffering sever aging, a complete SHM system is crucial to ensure the safety, serviceability, and sustainability of these large infrastructures. Preliminary results that were obtained in the first phase of the GeoSHM Demo Project showed the huge potential of the state-of-the-art Earth Observation in offering a better understanding of ground movements surrounding bridge sites. More importantly, the key to the successful SHM of long-span bridges is an innovative data strategy and reliable and affordable SHM sensor systems. Since the bridge response and modal frequencies are significantly influenced by wind, environmental factors, operational conditions, and ground movements, the data strategy must include smart data analysis to quantify effects from each external component, to offer further understanding of bridge behaviour and to produce reliable predictions and warnings. These are the aims and objectives of the GeoSHM Data Strategy, which will be focused and implemented on the FRB, as well as Erqi and Zhixi Bridges in the next phase of the GeoSHM Demon Project. Further discussion and results on analysing the monitoring data will be presented in separate papers.

## Figures and Tables

**Table 1:** Details of sensors to be installed during the GeoSHM Demo Project.

Sensors	Details	Sampling Rates (Hz)
GNSS	Leica GR10	10
GNSS	Panda DB38	1
Anemometer	Gill WindMaster	20
Weather Station	Gill MetPak	1
Accelerometer	Sherborne A545-0003-2G	100
Inclinometer	Sherborne LSOP-1	10
InSAR image	EO	1 image/14 days
